# Validation of *Trypanosoma cruzi* inactivation techniques for laboratory use

**DOI:** 10.1371/journal.pone.0300021

**Published:** 2024-04-18

**Authors:** Lorna M. MacLean, Mark Ariyanayagam, Lalitha Sastry, Christy Paterson, Manu De Rycker, Alan H. Fairlamb

**Affiliations:** 1 Drug Discovery Unit, Wellcome Centre for Anti-Infectives Research, School of Life Sciences, University of Dundee, Dundee, United Kingdom; 2 Division of Biological Chemistry and Drug Discovery, School of Life Sciences, University of Dundee, Dundee, United Kingdom; Kerman University of Medical Sciences, ISLAMIC REPUBLIC OF IRAN

## Abstract

*Trypanosoma cruzi* (*T*. *cruzi)* is the causative agent of Chagas’ disease, a parasitic infection responsible for significant morbidity and mortality in Latin America. The current treatments have many serious drawbacks and new drugs are urgently required. In the UK, *T*. *cruzi* is classified by the Advisory Committee on Dangerous Pathogens (ACDP) as a Hazard Group 3 organism and strict safety practices must be adhered to when handling this pathogen in the laboratory. Validated inactivation techniques are required for safe *T*. *cruzi* waste disposal and removal from Containment Level 3 (CL3) facilities for storage, transportation and experimental analysis. Here we assess three *T*. *cruzi*. inactivation methods. These include three freeze-thaw cycles, chemical inactivation with Virkon disinfectant, and air drying on Whatman FTA cards (A, B, C, Elute) and on a Mitra microsampling device. After each treatment parasite growth was monitored for 4–6 weeks by microscopic examination. Three freeze-thaw cycles were sufficient to inactivate all *T*. *cruzi* CLBrener Luc life cycle stages and Silvio x10/7 A1 large epimastigote cell pellets up to two grams wet weight. Virkon treatment for one hour inactivated *T*. *cruzi* Silvio x10/7 subclone A1 and CLBrener Luc both in whole blood and cell culture medium when incubated at a final concentration of 2.5% Virkon, or at ≥1% Virkon when in tenfold excess of sample volume. Air drying also inactivated *T*. *cruzi* CLBrener Luc spiked blood when dried on FTA A, B or Elute cards for ≥30 minutes and on a Mitra Microsampler for two hours. However, *T*. *cruzi* CLBrener Luc were not inactivated on FTA C cards when dried for up to two hours. These experimentally confirmed conditions provide three validated *T*. *cruzi* inactivation methods which can be applied to other related ACDP Hazard Group 2–3 kinetoplastid parasites.

## Introduction

*Trypanosoma cruzi* (*T*. *cruzi*) are flagellated protozoan parasites responsible for Chagas’ disease, also known as American trypanosomiasis. *T*. *cruzi* are transmitted to humans predominantly by the faeces of infected bloodsucking triatomine bugs at the bite site, through mucosal surfaces or broken skin. *T*. *cruzi* transmission can also occur congenitally, via organ transplantation, blood transfusion, or by ingestion of parasite contaminated food and drink [1, 2]. Cases of accidental laboratory-acquired infection have also been reported [3]. Chagas’ disease is endemic in Latin America with an estimated 8 million people infected resulting in 12,500 deaths per year [1, 4]. Chagas’ disease has an initial acute phase followed by a chronic phase which is life-long. Thirty percent of cases display severe clinical pathologies including cardiac problems and digestive disorders [5, 6]. The current treatments (benznidazole and nifurtimox) were developed over four decades ago and have many serious drawbacks [7, 8]. New treatments are urgently required; this has led to concerted research efforts to gain a better understanding of *T*. *cruzi* biology and Chagas’ disease pathogenesis as well as drug discovery and development. However, working with *T*. *cruzi* in research settings is challenging as it is classified as an ACDP Hazard Group 3 organism in the UK [9] and a Risk Group 3 in the EU [10], requiring cultivation in a Containment Level 3 (CL3) facility and adherence to a stringent code of practice that takes account of the nature of particular procedures and of the quantity of the agent involved (the USA, Canada, Australia and New Zealand categorize *T*. *cruzi* as Risk Group 2) [11]. The United Kingdom Health and Safety Executive (HSE) require validated *T*. *cruzi* inactivation methods for safe waste disposal within the CL3 facility and for safe removal of *T*. *cruzi* infected samples from the CL3 facility for storage, transportation and experimental analysis. Although many organisations will have established their own inactivation methods, limited evidence of their effectiveness against *T*. *cruzi* parasites is available in the literature. Here we assess three inactivation techniques for *T*. *cruzi* spiked mouse blood and cell culture samples including three freeze-thaw cycles, chemical inactivation with Virkon disinfectant and air drying on Whatman FTA cards (FTA A, B, C and Elute) and a Mitra microsampling device.

## Methods

### Cell culture

*T*. *cruzi* strain CL Brener expressing pTRIX2-PpyRE9h red-shifted firefly luciferase (CL Brener Luc) [12] known to be a discrete typing unit (DTU) TcVI strain, and Silvio X10/7 subclone A1 strain [13] (DTU TcI) were maintained as epimastigotes at 28ºC in RTH media [RPMI1640 (Sigma) supplemented with 0.4% trypticase peptone, 0.017 M HEPES, 25 μM haemin and 10% heat inactivated foetal calf serum (FCS)] [14–16].

CL Brener Luc trypomastigotes and intracellular amastigote cultures were obtained as previously described [17]. Briefly, 10^6^ ml^-1^ epimastigotes were incubated in RTH at 28ºC for 10 days and subsequently used to infect a monolayer of Vero cells (African green monkey kidney cells, ECCAC 84113001) at a multiplicity of infection (MOI) of 10 overnight at 37ºC 5% CO_2_ in Dulbecco’s modified Eagles’s medium (Lonza) supplemented with 10% FCS. Extracellular parasites were removed by washing with serum-free DMEM three times, followed by addition of fresh complete DMEM. *T*. *cruzi* infected Vero flasks were maintained at 37ºC 5% CO_2_ and DMEM replaced every 2–3 days until trypomastigotes emerged after ~6–7 days.

### Three freeze-thaw cycles

#### Epimastigotes in blood

Tissue culture derived CL Brener Luc epimastigotes were collected by centrifugation at 2000 × g for 10 min. Cell culture supernatant was removed and parasites resuspended at 10^8^ ml^-1^ in BALB/c mouse blood collected in an EDTA vacutainer. We also assessed the effect of three freeze-thaw cycles on 10 μl of mouse blood containing CL Brener Luc epimastigotes (10^6^) diluted with 20 μl sterile H_2_O (typical of a sample for mass spectrometry analysis). These were set-up in triplicate and subjected to three rapid snap freeze-thaw cycles by submerging in liquid nitrogen for 10 s followed by thawing at room temperature for 5 min until three cycles were completed. Subsequently samples were dispensed into a 24 well plate with 1 ml RTH media. Untreated epimastigote samples were included as controls. *T*. *cruzi* inactivation by this method was assessed by microscopic examination over 28 days for presence of motile parasites. *T*. *cruzi* were counted using a Neubauer haemocytometer after fixation 1:1 in 1% formaldehyde/PBS [limit of quantitation ≥2x10^4^ ml^-1^].

Three rapid freeze-thaw cycles were also performed as described above on 100 μl of undiluted mouse blood spiked with 10^7^ CL Brener Luc epimastigotes to determine the effect of this method on a larger volume of blood not under hypo-osmotic stress. This sample was placed in a T25 flask with 10 ml RTH media, incubated at 28ºC, and parasites were counted at day 28.

#### Epimastigote pellets

Three freeze-thaw cycles were assessed as a method to inactivate large cell pellets (2x10^10^ epimastigotes) using a dry ice bath for 10 min per step. Silvio X10/7 subclone A1 epimastigotes were grown in RTH in 1 l roller bottles for 3–4 d to a density of 2-4x10^7^ ml^-1^ at 28ºC. Parasites were harvested by centrifugation at 900 × g for 35 min, supernatant removed, parasites resuspended in 50 ml PBS, counted and centrifuged at 900 × g for 15 min. The supernatant was aspirated to yield large cell pellets of 2x10^10^ parasites in 50 ml falcon tubes. These were submerged in a dry ice bath (dry ice pellets plus isopropanol) for 10 min and subsequently thawed for 10 min in a water bath at room temperature; this was repeated three times. Upon each thawing the pellets were visually inspected to ensure they were completely thawed. *T*. *cruzi* epimastigote inactivation by this method was assessed by placing ~10% (measured by weight) of each pellet in a T175 flask with 50 ml RTH for 6 weeks at 28ºC and monitoring weekly for presence of motile epimastigotes by microscopic examination. A control T175 flask was set-up with 10 epimastigotes to determine how long it would take to clearly observe motile parasites at a very low seeding density. In addition, a flask was set-up using the same batch of RTH media at a 1000-fold parasite dilution of the starting flask to demonstrate media competence. This process was performed on 43 sample pellets.

#### Trypomastigotes

The rapid freeze-thaw protocol in liquid nitrogen described above for epimastigotes in blood was also performed using CL Brener Luc trypomastigotes. Briefly, cell culture-derived trypomastigotes were harvested from the supernatant of infected Vero cell cultures after egress, centrifuged and resuspended in mouse blood at 10^8^ ml^-1^. Sterile water (20 μl) was added to 10 μl of mouse blood (BALB/c) containing 10^6^ CL Brener Luc trypomastigote parasites, set-up in triplicate and three rapid freeze-thaw cycles completed. As *T*. *cruzi* trypomastigotes are non-replicative forms, these samples were placed on a Vero cell monolayer (10^5^ cells) plus 1 ml DMEM/10% FCS in 24 well plates overnight. They were subsequently washed with DMEM three times to remove any extracellular parasites and replenished with 1 ml DMEM/1% FCS twice weekly. Viable trypomastigotes can establish Vero cell infection, transform into intracellular replicating amastigotes and emerge from Vero cells as motile trypomastigotes, thus, presence of motile trypomastigotes was used to assess this inactivation method by microscopic examination over 28 days. Vero cells have a doubling time of ~24 h and are typically sub-cultured 2–3 times per week. In order to maintain cells during the 4 week monitoring period without sub-culture all Vero cell cultures were set-up in 1% FCS to reduce the Vero cell replication rate [18]. Untreated mouse blood spiked with trypomastigotes (10 μl plus 20 μl H_2_O) were used as controls to determine Vero cell cycling time of CL Brener Luc under these conditions.

Three rapid freeze-thaw cycles were also carried out on 100 μl of mouse blood spiked with 10^7^ *T*. *cruzi* trypomastigotes to assess this method on a larger volume of blood not under osmotic stress. Treated and untreated samples were incubated overnight with a Vero cell monolayer (10^6^ cells) in T25 flasks, DMEM/10% FCS at 37ºC 5% CO_2_. Extracellular parasites were removed the following day as described above. Flasks were maintained at 37ºC 5% CO_2_ in DMEM/1% FCS and media was replaced twice weekly. This assay was performed in triplicate and cultures were monitored for the emergence of parasites for 28 days.

#### Intracellular amastigotes

*T*. *cruzi* CL Brener Luc infected Vero cells were harvested after 5 days infection (multiplicity of infection of 10). Cell culture supernatant was aspirated, Vero cells washed with DMEM without FCS three times followed by 5 min incubation with Trypsin-EDTA (Gibco). After addition of DMEM/10% FCS, cells were resuspended at 10^6^ ml^-1^ in triplicate and samples (1 ml) subjected to three rapid freeze-thaw cycles as described above. Treated and untreated samples were incubated with 10^6^ Vero cells in T25 flasks, DMEM/10% at 37ºC 5% CO_2_ overnight. Flasks were subsequently washed and maintained as described above in DMEM/1% FCS. Vero cell cultures were monitored for the emergence of trypomastigotes for 28 days.

### Chemical inactivation- Virkon

#### Epimastigotes in blood and cell culture media

For qualitative experiments, *T*. *cruzi* Silvio X10/7 subclone A1 epimastigotes (5x10^7^) suspended in 1 ml rat blood were mixed with 1 volume of 1% Virkon (w/v) solution, sterile tap water (0.2 micron filtered) or PBS. After incubation for 1 h at room temperature, samples were centrifuged to pellet parasites, washed twice with PBS and re-suspended in 1 ml RTH/FCS. Parasites were then serially diluted tenfold into 24 well plates at 10^7^ to 10^1^/ml in RTH/FCS, incubated at 28°C and microscopically examined for motile parasites on day 1, 8, 15, 20, 25 and 32.

For quantitative experiments, CL Brener Luc epimastigotes were centrifuged at 2000 × g 10 min, supernatant removed, washed in PBS and re-suspended at 2x10^7^ ml^-1^ in mouse blood (C57/BL6) and in RTH (10% FCS) media. Samples (100 μl, in triplicate) were mixed with 100 μl and 900 μl of 1%, 2% and 5% Virkon solution (Rely+On^™^VIRKON^™^, LanXESS Corporation) and PBS. Samples were incubated for 1 h at room temperature, subsequently centrifuged, washed twice with PBS and re-suspended in 1 ml RTH. These were dispensed into 24-well plates, incubated at 28 ºC 5% CO_2_ and monitored for the presence of motile parasites over 28 days by microscopic examination. Parasite density was determined using a Neubauer haemocytometer as described above.

### Drying techniques

#### Epimastigotes in blood- Mitra microsampling device

A Mitra Microsampler (Phenomenex) [19] was used to collect mouse blood (10 μl) spiked with 10^8^ ml^-1^ CL Brener Luc epimastigote parasites in triplicate. Samples were left to dry at room temperature for 2 h. The tip containing the sample was subsequently added to a 24 well plate with 1 ml RTH and incubated at 28 ºC 5% CO_2_. Epimastigote inactivation by this drying method was determined by monitoring cultures for presence of motile parasites by microscopic examination over 28 days.

#### Epimastigotes in blood- Whatman^®^ FTA^®^ cards

Whatman FTA DMPK A, B, C and Elute cards (GE Healthcare Life Sci) were sterilised prior to use by lightly spraying with 70% ethanol and air drying. The sample application area was cut out and placed into a 24 well plate. Mouse blood (20 μl) containing 10^8^ ml^-1^ CL Brener Luc epimastigotes was spotted onto each card and allowed to air dry for 15, 30, 60 and 120 min before addition of 1 ml RTH. Wells were monitored for presence of motile parasites for 28 days by microscopic examination. This assay was performed in duplicate for each time point.

## Results

### Three freeze-thaw cycles

#### Epimastigotes in blood

**Fig 1:**
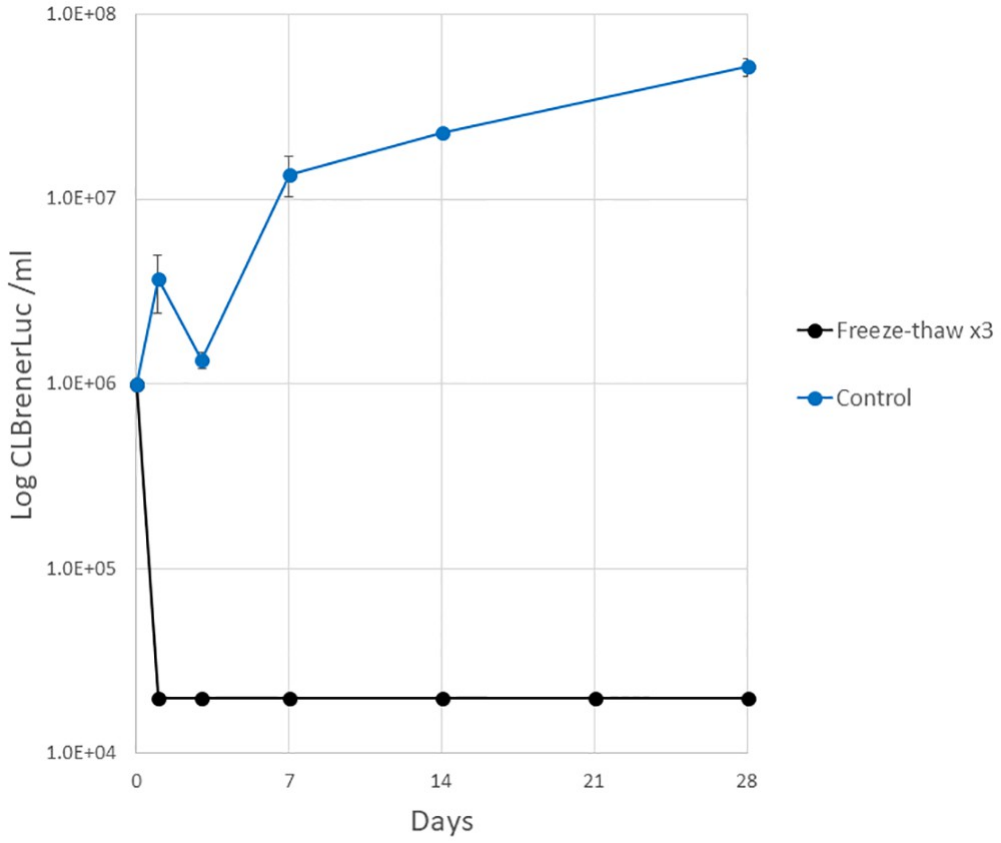
*T*. *cruzi* epimastigote growth after three rapid freeze-thaw cycles. Samples of mouse blood spiked with *T*. *cruzi* CLBrener Luc were subjected to three freeze-thaw cycles and monitored for parasite growth over 28 days (three technical replicates, mean ± standard deviation, limit of quantitation 2×10^4^ ml^-1^).

Pharmacokinetic studies play a pivotal role in drug development; these include mass spectrometry analysis of blood samples taken from animal models and typically require dilution in water prior to evaluation. We therefore assessed the effect of three freeze-thaw cycles on 10 μl of mouse blood containing CL Brener Luc epimastigotes (10^6^) diluted with sterile H_2_O (20 μl). Three rapid freeze-thaw cycles successfully inactivated CL Brener Luc epimastigotes blood / water samples with no motile parasites observed after 28 days in culture. Epimastigote growth was recorded in the spiked blood / water controls (Fig 1, S1 Table). Three rapid freeze-thaw cycles were also sufficient to inactivate undiluted epimastigote (10^7^) spiked blood samples with no motile parasites detected in cultures over 28 days (S1 Table).

#### Epimastigote pellets

Large Silvio X10/7 subclone A1 epimastigote pellets (2x10^10^) were inactivated in 41 of 43 samples by three 10 min freeze-thaw cycles with no motile parasites observed after 6 weeks in culture. Epimastigotes were generally visible by day 7 in the very low seeding density control flasks with media competence confirmed in all runs. It was noted that the average weight of pellets was 1.3 g. However, the two pellets that failed the inactivation procedure weighed 2.3 and 3.1 g despite the same parasite numbers. The increased weight is likely due to insufficient removal of supernatant after centrifugation. It is, therefore, important to remove as much supernatant as possible before performing freeze-thaw cycles. This inactivation method is validated for *T*. *cruzi* Silvio X10/7 subclone A1 pellets weighing ≤2 g, longer duration freeze thaw cycles may be required for larger pellets. In addition, in previous experiments when large pellets (≤2 g) were frozen on dry ice without isopropanol, 1 out of 12 failed inactivation, possibly due to less efficient heat transfer in solid CO_2_.

#### Trypomastigotes

Three rapid freeze-thaw cycles inactivated CL Brener Luc trypomastigote (10^6^) spiked blood / water samples with no motile parasites observed emerging from Vero cells after 28 days (Table 1). In control samples trypomastigotes were detected after 10 days incubation with Vero cells confirming media and cell viability.

**Table 1:** Effect of freeze thawing on survival of *T*. *cruzi* trypomastigote and amastigote stages. CL Brener Luc trypomastigote spiked mouse blood and *T*. *cruzi*-infected Vero cells were subjected to three freeze-thaw cycles (F-T), before being incubated with fresh Vero cells and monitored for trypomastigote emergence over 28 days. Three technical replicates, mean (standard deviation).

Sample	CL Brener Luciferase Trypomastigote Counts ml^-1^								
	Day								
	0	1	3	7	10	14	21	24	28
F-T Spiked blood + H_2_O	10^6^	-/-/-	-/-/-	-/-/-	-/-/-	-/-/-	-/-/-	-/-/-	-/-/-
F-T Spiked blood	10^7^	-/-/-	-/-/-	-/-/-	-/-/-	-/-/-	-/-/-	-/-/-	-/-/-
F-T Infected Vero cells	*	-/-/-	-/-/-	-/-/-	-/-/-	-/-/-	-/-/-	-/-/-	-/-/-
Control Spiked blood + H_2_O	10^6^	-/-/-	-/-/-	-/-/-	+/+/+	3x10^4^ (2x10^4^)	3x10^4^ (1x10^4^)	nd	nd
Control Spiked blood	10^7^	-/-/-	-/-/-	+/+/+	2x10^4^ (0)	2x10^5^ (6x10^4^)	nd	nd	nd
Control Infected Vero Cells	*	-/-/-	-/-/-	+/+/+	3x10^5^ (2x10^4^)	nd	nd	nd	nd

* Infected Vero cells were harvested after 5 days *T*. *cruzi* infection (MOI10), resuspended at 10^6^ ml^-1^, freeze-thawed and control samples were subsequently incubated with 10^5^ ml^-1^ Vero cells

+/+/+ Motile trypomastigotes observed in all three replicates, but below limit of quantitation 2x10^4^ ml^-1^

-/-/- No motile trypomastigotes observed in all three replicates

nd Not done

Three rapid freeze-thaw cycles were also sufficient to inactivate 100 μl trypomastigote (10^7^) undiluted spiked blood samples. No motile trypomastigotes emerged from Vero cells after 28 days (Table 1). In control samples trypomastigotes started to emerge from Vero cells at day 7. Growth of Vero cells plus 100 μl blood (not spiked) in 1% FCS was recorded at day 0 (10^5^ ml^-1^) to day 28 (2x10^6^ ml^-1^, doubling time 6 days). This is in line with reduced Vero cell growth under low FCS conditions suggesting whole mouse blood did not adversely affect Vero cells.

#### Intracellular amastigotes

Three rapid freeze-thaw cycles inactivated intracellular *T*. *cruzi* CLBrener Luc. No parasites emerged from Vero cells infected with freeze-thawed *T*. *cruzi* infected Vero cells after 4 weeks, while trypomastigotes started to emerge from Vero cells at day 7 in the untreated control cultures (Table 1).

### Chemical inactivation- Virkon

Virkon is a disinfectant containing an oxidising agent (potassium peroxymonosulfate), a detergent (sodium dodecylbenzenesulfonate) and organic acids (sulfamic acid and malic acid) plus sodium hexametaphosphate to buffer at low pH [20]. Preliminary experiments with Virkon showed that exposure to a 0.5% solution for 10 min was unable to kill all *T*. *cruzi* Silvio X10/7 subclone A1 epimastigotes in whole blood or serum as assessed by microscopic examination for motile parasites. Consequently, all subsequent experiments involved a 1 h exposure. Parasites (10^7^ ml^-1^) in whole blood or whole serum also survived exposure for 1 h in a 1:1 mixture with 1% Virkon likely due to the neutralising effect of the high organic matter on Virkon’s active components. A subsequent experiment showed that heavily infected whole blood or serum samples must be diluted ten-fold with 1% Virkon for 1 h for complete inactivation (Table 2). Using a ten-fold dilution of the treated sample into fresh medium it is possible to assess the log reduction in viable parasites following long-term culture. Viable parasites could be detected in PBS control cultures down to a dilution of 10^2^ ml^-1^ from 15 days onwards, whereas no motile parasites were detected in Virkon-treated blood after 32 days with an initial inoculum of 10^7^ ml^-1^. This equates to a 5 log (10^5^-fold) effective kill. African trypanosomes are considered to be particularly susceptible to osmotic shock, but a 1 h exposure to tap water (10:1) has only a 2 log (100-fold) effective kill in the case of *T*. *cruzi* (Table 2).

**Table 2:** Effect of Virkon treatment on *T*. *cruzi* epimastigote viability in rat blood. *T*. *cruzi* strain Silvio X10/7 subclone A1 infected rat blood, was treated for 1 h with a 10-fold (v/v) excess of 1% Virkon. Samples were pelleted and resuspended in the same volume of culture medium before 10-fold dilution into culture medium. Viable parasites were detected by microscopic examination for motile epimastigotes and scored qualitatively. Parasite positive samples are highlighted as a heat map.

*T*. *cruzi* infected sample	Treatment	Day	Silvio X10/7 A1 Starting Density, cells ml^-1^						
			10^7^	10^6^	10^5^	10^4^	10^3^	10^2^	10^1^
Rat blood	PBS control	8	NV	++	+++	++	+	-	-
		15	++	++++	+++	++	++	+	-
		20	++	++++	++++	+++	+	+	-
		25	+++	++++	++++	+++	++	+	-
		32	-	+++	++++	++++	++++	-	-
Rat blood	Water	8	-	-	-	-	-	-	-
		15	-	-	-	-	-	-	-
		20	+/-	+	+	+	-	-	-
		25	++	++	++	+	-	-	-
		32	-	-	+	+	-	-	-
Rat blood	1% Virkon	8	NV	-	-	-	-	-	-
		15	NV	-	-	-	-	-	-
		20	NV	-	-	-	-	-	-
		25	NV	-	-	-	-	-	-
		32	NV	-	-	-	-	-	-

Only motile parasites scored as ‘+’

NV = Suspension too thick to detect motile parasites

- no motile parasites seen in ≥20 fields

+<1 per field (20 fields searched)

++ 1–5 per field

+++ >5 per field

++++ Numerous parasites with rosette formation

**Fig 2:**
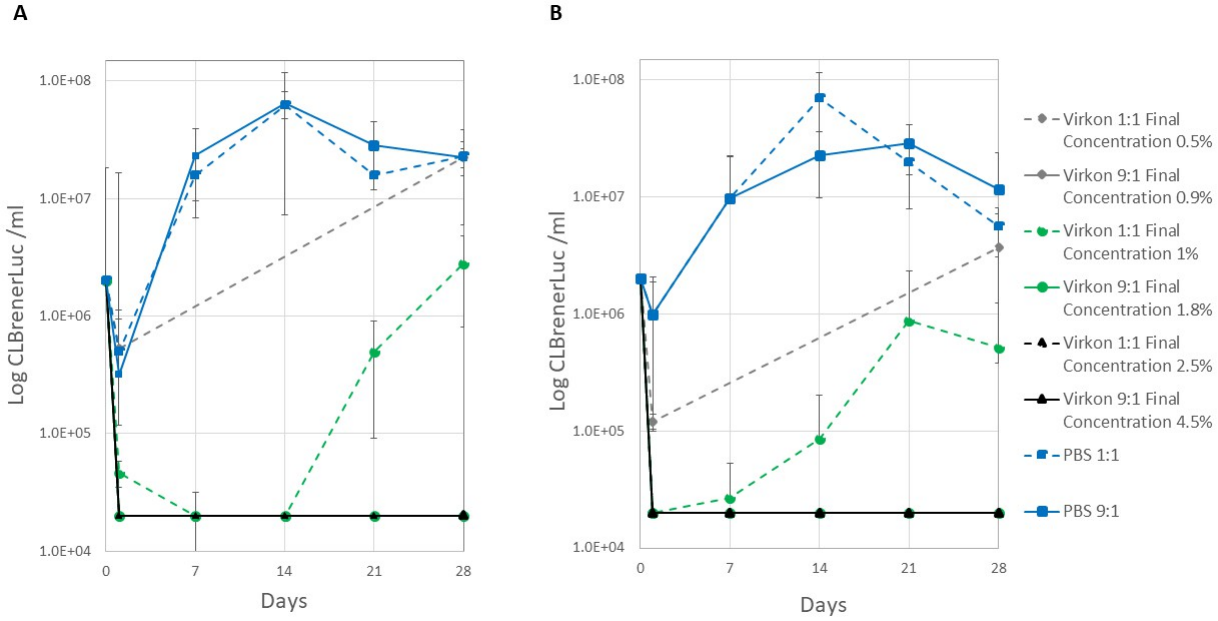
Virkon inactivation of *T*. *cruzi* epimastigotes in blood (A) and RTH media (B). *T*. *cruzi* CLBrener Luc epimastigotes were incubated for 1 h with 1, 2 and 5% Virkon 1:1 (dashed lines) and 9:1 (solid lines) volumetric excess giving a final Virkon concentration range of 0.5–4.5%. Cultures were monitored for parasite growth over 28 days (three technical replicates, mean ± standard deviation, limit of quantitation 2e4 ml^-1^). PBS controls were included.

Further quantitative experiments were carried out with *T*. *cruzi* CLBrener Luc epimastigotes (2x10^6^) in whole mouse blood and cell culture media (RTH 10% FCS). These confirmed that parasites were not inactivated by 1 h treatment 1:1 with 1% Virkon (final concentration 0.5% w/v) in whole blood. Although a 2- and 8-fold decrease in parasite counts was observed at day 1 in spiked blood and in RTH samples, respectively, parasite growth was recorded at day 28 (Fig 2A and 2B). A larger decrease in parasite counts was observed in spiked blood samples following 1 h treatment 1:1 with 2% Virkon at day 1 (21-fold) that declined to below the limit of quantitation at day 7 and 14. However, motile parasites were observed from day 21 (Fig 2A and 2B). In *T*. *cruzi* cell culture samples no motile parasites were observed on day 1 after treatment at a final concentration of 1% Virkon, however, growth was observed from day 7 (Fig 2B). Treatment for 1 h with 5% Virkon at a ratio 1:1 (final concentration 2.5%) successfully inactivated both *T*. *cruzi* spiked blood and RTH samples (Fig 2A and 2B). When spiked blood and RTH samples were treated for 1 h with a 9-fold excess volume of Virkon (final concentration 0.9, 1.8 and 4.5% Virkon) no viable parasites were detected over 28 days at all 3 concentrations tested (Fig 2A and 2B). Epimastigote growth was observed in all PBS treated controls.

### Drying techniques

Air drying *T*. *cruzi* CLBrener Luc spiked blood (10^6^) for 2 h after collection using the Mitra Microsampler was sufficient to inactivate these samples, with no motile epimastigotes detected in cultures up to 28 days (S2 Table).

**Fig 3:**
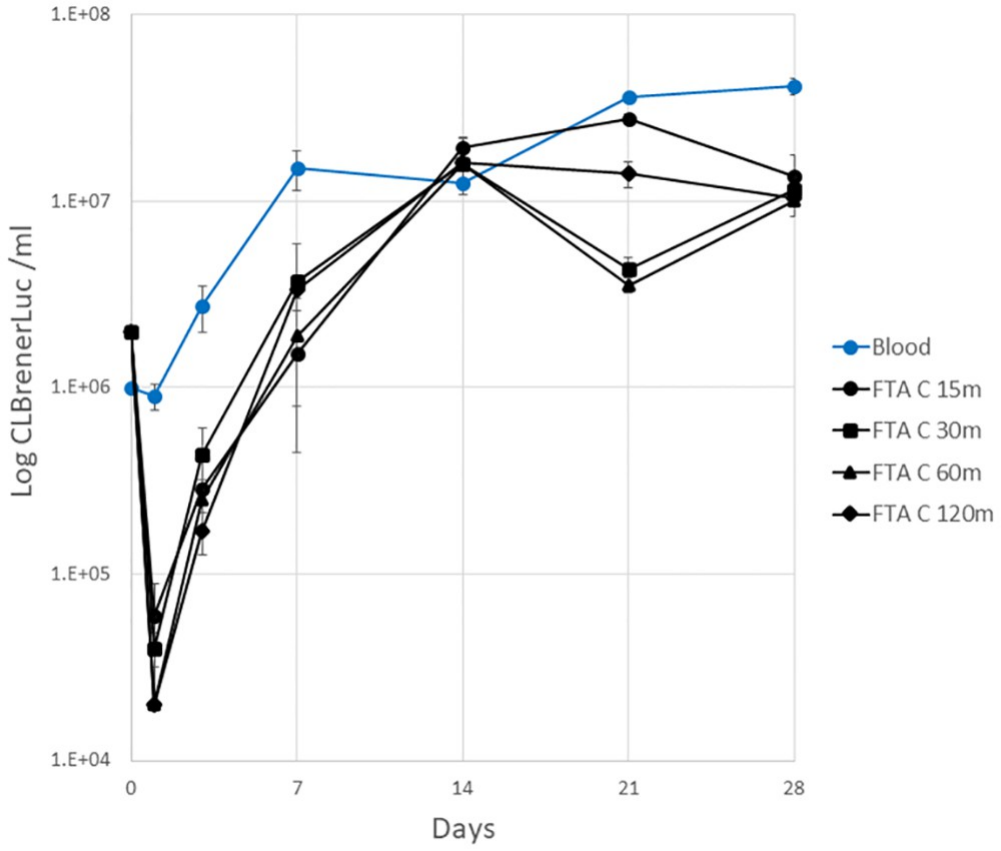
*T*. *cruzi* epimastigote growth after air drying on FTA C cards. CL Brener Luc epimastigote spiked mouse blood (0.02 ml) spotted on FTA C cards and air dried for 15–120 min. *T*. *cruzi* infected blood control (not dried) was also included and parasite growth recorded over 28 days (mean ±SD, limit of quantitation 2×10^4^ ml^-1^).

No motile *T*. *cruzi* were observed in cultures of epimastigote spiked blood (2x10^6^) collected on FTA A and B cards and dried for 15 to 120 min (S2 Table). One parasite was detected by microscopic examination of the well of one replicate after 15 min drying on FTA Elute cards at day 1, 3 and 7 but was not detected at day 14 to 28 of cell culture. No parasites were detected in FTA Elute card samples up to 28 days in culture when dried for 30, 60 or 120 min (S2 Table). However, for all drying times (15 to 120 min), motile parasites were detected in FTA C card cultures at all timepoints of the monitoring period (Fig 3, S2 Table).

## Discussion

Many laboratories use disinfectants and autoclaving as methods to inactivate waste contaminated with human and animal pathogens. It is required that these methods are validated for efficacy against pathogens before work is permitted. Published articles in this area are limited with most laboratories performing validation experiments for the pathogens they work on without publishing the data. We have now successfully validated three methodologies to inactivate *T*. *cruzi* in cell culture and blood samples. These include three freeze-thaw cycles, Virkon treatment (final concentration 2.5% for 1:1 dilutions, or ≥1% when Virkon solution is applied in 10-fold volumetric excess) and air drying on FTA A, B and Elute cards (≥30 min) or on a Mitra Microsampler (2 h).

We also established that three freeze-thaw cycles are capable of inactivating Silvio X10/7 A1 epimastigote pellets up to 2 g wet weight (~2x10^10^ parasites). Furthermore, three freeze-thaw cycles are effective against the more clinically relevant mammalian stages of the *T*. *cruzi* CLBrener Luc life cycle. Although both three rapid freeze-thaw cycles in liquid nitrogen and 10-min cycles in a dry ice/isopropanol bath were effective against *T*. *cruzi* epimastigotes, when inactivating large parasite pellets the combined weight of the pellet and remaining supernatant prior to freeze-thaw is critical and must be ≤2 g as two heavier pellets failed inactivation. It is therefore crucial that large parasite pellets are frozen and submerged in a dry ice/ isopropanol bath as performed in this study to ensure consistent heat transfer and complete freezing of the pellet and contact with the entire pellet containing tube. Furthermore, it is vital that a minimum of three freeze-thaw cycles are carried out. Previous work showed that when a thick suspension of 10^10^ *T*. *cruzi* Silvio X10/7 A1 epimastigotes was dispensed dropwise into liquid nitrogen to form ‘noodles’ for cryogrinding purposes, motile parasites could be recovered after 20 days in culture (RTH). Thus, one rapid freeze-thaw cycle is not sufficient to inactivate *T*. *cruzi* (S. Wyllie, personal communication).

A study assessing the ability of *T*. *cruzi* to survive the processing and storage conditions of human cells, tissues and tissue-based products found that *T*. *cruzi* trypomastigotes survived 24 h at room temperature, at least 10 days at 4ºC and for long periods as stabilates at -80 ºC [21]. They also showed that trypomastigotes in media or cryoprotectant were viable after 4 freeze-thaw cycles. However, in this case trypomastigotes were frozen in a Mr Frosty (Nalgene) giving a slow freezing rate of 1 ºC per min in a -80 ºC freezer and thawed at weekly intervals. This suggests that it is not only the number of freeze-thaw cycles, but also rapid freezing that is important causing rigid ice crystal formation damaging cell membranes during the freeze-thaw procedure leading to cell death.

Virkon was first developed in the 1980’s as a broad-spectrum disinfectant. It is a mix of six biocides acting in different ways against viruses and many bacteria and fungi [20, 22]. The principal active components are oxidising agents that combine to form hypochlorous acid and a detergent. The biocidal activity of both of these components is attenuated by the presence of organic material and this is borne out in the present study in that it is the ratio of blood or serum to Virkon that is critical for effective disinfection.

Whatman FTA DMPK-A, B and FTA Elute cards are cellulose paper impregnated with chemicals to lyse cells and denature proteins on contact. They are designed to inactivate organisms, including blood-borne pathogens, allowing safe transport and storage of samples at room temperature. FTA DMPK- A and B cards are typically used for analyte quantitation by HPLC-MS/MS, while FTA Elute cards capture nucleic acids on the matrix for downstream experiments. FTA DMPK-C cards can be used when the coating on FTA A and B cards interferes with analyte quantitation. However, FTA DMPK-C cards contain pure cellulose and are not impregnated with chemicals, meaning that air drying is the only method used for pathogen inactivation. Notably drying techniques were only effective at inactivating *T*. *cruzi* epimastigote infected blood when dried on FTA DMPK-A, B or Elute cards for ≥30 min. These cards are not visibly dry until they have been incubated at room temperature for 1 h, thus, it is recommended that *T*. *cruzi* samples collected on these cards are dried for a minimum of 1 h before removal from a biosafety facility. Motile epimastigotes were observed at all drying times on FTA DMPK- C cards, thus, 2 h air drying alone is not sufficient to kill *T*. *cruzi*. This may be due to insufficient drying time, but the cards were visibly dry and a prolonged drying period was not tested to confirm this. Although we have not assessed parasite viability beyond 2 h it is interesting to note that in a separate study *T*. *cruzi* trypomastigotes were still infective 14 days after *Triatoma infestans* vector death [23] highlighting the need for validation for specific inactivation methods. *T*. *cruzi* epimastigote infected blood was inactivated after 2 h air drying on a Mitra Microsampler. It is unclear what material this device is made of, but the manufacturers state it is a ‘hydrophilic porous material that rapidly wicks fluid and dries in 2 h or less’.

In this study we have used a common *in vitro T*. *cruzi* strain Silvio X10/7 A1 and animal model strain CLBrener Luc. Although it is unlikely resistance to inactivation techniques would vary between *T*. *cruzi* strains, it may be prudent for those using different strains or different experimental conditions to consider validation in a similar way as described here.

A previous study assessed the effect common laboratory disinfectants and 50 ºC heat on a range of trypanosomatid parasites including bloodstream form *T*. *brucei*, *T*. *rangeli* epimastigotes and *L*. *major* promastigotes [24]. After 5 min of each treatment, live cells were counted on a haemocytometer. Using this method, it was determined that all treatments were able to kill all three parasites stating a 100% lethal concentration of 0.05% for bleach, 0.2% for TriGene, 15–17.5% for ethanol, 0.1% for liquid hand soap and 80–90% for water alone suggesting these parasites do not endure hypo-osmotic stress. Furthermore, a report verified killing of *T*. *brucei* by 20-sec treatment with ≥0.1% TriGene and complete cell lysis at ≥1% TriGene [25]. Wang et al [24] propose that other trypanosomatids are likely to be susceptible to these inactivation methods including *T*. *cruzi* which, like *T*. *rangeli*, is a stercorarian trypanosome. However, our data has shown that even when very small numbers of *T*. *cruzi* survive (below the limit of quantitation), they are able to establish cultures with high parasite densities at later time points. In the case of *T*. *cruzi*, therefore, growth must be monitored for at least 4 weeks after treatment to ensure 100% killing. In addition, although *T*. *rangeli* were slightly more resistant to water inactivation than African trypanosomes, they still succumbed to treatment. Thus, *T*. *cruzi* appears to be considerably more robust than other kinetoplastid parasites, suggesting the successful inactivation methods validated here can be extrapolated to other kinetoplastid parasites.

## Conclusions

For successful *T*. *cruzi* inactivation the following methods have been validated:

1. Three consecutive freeze-thaw cycles on *T*. *cruzi* Silvio X10/7 A1 and CLBrener Luc
2. Incubation of *T*. *cruzi* Silvio X10/7 A1 and CLBrener Luc for 1 h with Virkon disinfectant at a final concentration of 2.5% or at a final concentration of ≥1% when in tenfold volumetric excess
3. Air drying *T*. *cruzi* CLBrener Luc infected blood samples on FTA cards A, B and Elute for ≥1 h
4. Air drying *T*. *cruzi* CLBrener Luc infected blood samples on a Mitra microsampling device for 2 h

### Notes

1. For *T*. *cruzi* Silvio X10/7 A1 epimastigote pellets >2 g three 10 min freeze cycles were not sufficient to achieve full inactivation
2. Air drying *T*. *cruzi* CLBrener Luc infected blood samples for 2 h on FTA DMPK-C cards was not sufficient to achieve inactivation

## Supporting information

S1 Data (XLSX)

S1 Table CL Brener Luciferase epimastigote growth after three rapid freeze-thaw (F-T) cycles. Mouse blood samples spiked with *T*. *cruzi* were subjected to three freeze-thaw cycles and monitored for parasite growth over 28 days. Three technical replicates, mean (standard deviation).
(DOCX)

S2 Table CL Brener Luciferase epimastigote growth after 15 min to 2 h drying spiked mouse blood on FTA cards. Parasite growth was monitored over 28 days, mean (standard deviation).
(DOCX)
