# Supplementary material for: Validation of Trypanosoma cruzi inactivation techniques for laboratory use
Source: PLoS One. 2024 Apr 18;19(4):e0300021. doi: 10.1371/journal.pone.0300021 (PMC11025933; doi:10.1371/journal.pone.0300021)
Supplement: S1 Table — Mouse blood samples spiked with T. cruzi were subjected to three freeze-thaw cycles and monitored for parasite growth over 28 days. Three technical replicates, mean (standard deviation). (DOCX) [file pone.0300021.s002.docx]

**Table S1. CL Brener Luciferase epimastigote growth after three rapid freeze-thaw (F-T) cycles.** Mouse blood samples spiked with *T. cruzi* were subjected to three freeze-thaw cycles and monitored for parasite growth over 28 days. Three technical replicates, mean (standard deviation)**.**

| **Sample** | **CL Brener Luciferase Epimastigote Counts ml^-1^** | | | | | | |
| --- | --- | --- | --- | --- | --- | --- | --- |
|  | **Day** | | | | | | |
|  | 0 | 1 | 3 | 7 | 14 | 21 | 28 |
| F-T  Spiked blood + H_2_O | 10^6^ | -/-/- | -/-/- | -/-/- | -/-/- | -/-/- | -/-/- |
| F-T  Spiked blood | 10^7^ | nd | nd | nd | nd | nd | -/-/- |
| Control  Spiked blood + H_2_O | 10^6^ | 4x10^6^  (1x10^6^) | 1x10^6^  (1x10^5^) | 1x10^7^  (3x10^6^) | 2x10^7^  (4x10^5^) | nd | 5x10^7^  (6x10^6^) |

-/-/- No motile trypomastigotes observed in all three replicates
