# Supplementary material for: Validation of Trypanosoma cruzi inactivation techniques for laboratory use
Source: PLoS One. 2024 Apr 18;19(4):e0300021. doi: 10.1371/journal.pone.0300021 (PMC11025933; doi:10.1371/journal.pone.0300021)
Supplement: S2 Table — Parasite growth was monitored over 28 days, mean (standard deviation). (DOCX) [file pone.0300021.s003.docx]

**Table S2. CL Brener Luciferase epimastigote growth after 15 min to 2 h drying spiked mouse blood on FTA cards and a Mitra microsampling device (Mitra).** Parasite growth was monitored over 28 days, mean (standard deviation)**.**

| **Drying Treatment** | **CL Brener Luciferase Epimastigote Counts ml^-1^** | | | | | | |
| --- | --- | --- | --- | --- | --- | --- | --- |
|  | **Day** | | | | | | |
|  | **0** | **1** | **3** | **7** | **14** | **21** | **28** |
| **FTA A** |  |  |  |  |  |  |  |
| 15 min | 2x10^6^ | -/-/- | -/-/- | -/-/- | -/-/- | -/-/- | -/-/- |
| 30 min | 2x10^6^ | -/-/- | -/-/- | -/-/- | -/-/- | -/-/- | -/-/- |
| 60 min | 2x10^6^ | -/-/- | -/-/- | -/-/- | -/-/- | -/-/- | -/-/- |
| 120 min | 2x10^6^ | -/-/- | -/-/- | -/-/- | -/-/- | -/-/- | -/-/- |
| **FTA B** |  |  |  |  |  |  |  |
| 15 min | 2x10^6^ | -/-/- | -/-/- | -/-/- | -/-/- | -/-/- | -/-/- |
| 30 min | 2x10^6^ | -/-/- | -/-/- | -/-/- | -/-/- | -/-/- | -/-/- |
| 60 min | 2x10^6^ | -/-/- | -/-/- | -/-/- | -/-/- | -/-/- | -/-/- |
| 120 min | 2x10^6^ | -/-/- | -/-/- | -/-/- | -/-/- | -/-/- | -/-/- |
| **FTA C** |  |  |  |  |  |  |  |
| 15 min | 2x10^6^ | 6x10^4^  (3x10^4^) | 3x10^5^  (2x10^4^) | 2x10^6^  (10^6^) | 2x10^7^  (3x10^6^) | 3x10^7^  (2x10^6^) | 10^7^  (4x10^6^) |
| 30 min | 2x10^6^ | 4x10^4^  (0) | 4x10^5^  (2x10^5^) | 4x10^6^  (2x10^6^) | 2x10^7^  (3x10^6^) | 4x10^6^  (6x10^5^) | 10^7^  (8x10^5^) |
| 60 min | 2x10^6^ | 2x10^4^  (0) | 3x10^5^  (7x10^4^) | 2x10^6^  (10^6^) | 2x10^7^  (3x10^5^) | 4x10^6^  (4x10^5^) | 10^7^  (2x10^6^) |
| 120 min | 2x10^6^ | 2x10^4^  (3x10^4^) | 2x10^5^  (4x10^4^) | 3x10^6^  (9x10^4^) | 2x10^7^  (5x10^6^) | 1x10^7^  (2x10^6^) | 1x10^7^  (0) |
| **FTA Elute** |  |  |  |  |  |  |  |
| 15 min | 2x10^6^ | + | + | + | -/-/- | -/-/- | -/-/- |
| 30 min | 2x10^6^ | -/-/- | -/-/- | -/-/- | -/-/- | -/-/- | -/-/- |
| 60 min | 2x10^6^ | -/-/- | -/-/- | -/-/- | -/-/- | -/-/- | -/-/- |
| 120 min | 2x10^6^ | -/-/- | -/-/- | -/-/- | -/-/- | -/-/- | -/-/- |
| **Mitra**  120 min | 10^6^ | -/-/- | -/-/- | -/-/- | -/-/- | -/-/- | -/-/- |

+ 1 epimastigote observed in 1 well but below the limit of quantitation 2x10^4^ ml^-1^ and not observed in the other replicates

-/-/- No motile trypomastigotes observed in all three replicates
